# Maternal mortality in South Africa in 2001: From demographic census to epidemiological investigation

**DOI:** 10.1186/1478-7954-6-4

**Published:** 2008-08-21

**Authors:** Michel Garenne, Robert McCaa, Kourtoum Nacro

**Affiliations:** 1 French Institute for Research and Development (IRD) and Institut Pasteur, Paris, France; 2 Minnesota Population Center, University of Minnesota, USA, and Coordinator of the IPUMS-International project, USA; 3 United Fund for Population Activities (UNFPA), New York, USA

## Abstract

**Background:**

Maternal mortality remains poorly researched in Africa, and is likely to worsen dramatically as a consequence of HIV/AIDS.

**Methods:**

The 2001 census of South Africa included a question on deaths in the previous 12 months, and two questions on external causes and maternal mortality, defined as "pregnancy-related deaths". A microdata sample from the census permits researchers to assess levels and differentials in maternal mortality, in a country severely affected by high death rates from HIV/AIDS and from external causes.

**Results:**

After correcting for several minor biases, our estimate of the Maternal Mortality Ratio (MMR) in 2001 was 542 per 100,000 live births. This level is much higher than previous estimates dating from pre-HIV/AIDS times. This high level occurred despite a relatively low proportion of maternal deaths (6.4%) among deaths of women aged 15–49 years, and was due to the astonishingly high level of adult mortality, some 4.7 times higher than expected from mortality below age 15 or above age 50. The main reasons for these excessive levels were HIV/AIDS and external causes of deaths. Our regional estimates of MMR were found to be consistent with other findings in the Cape Town area, and with the Agincourt DSS. The differentials in MMR were considerable: 1 to 9.2 for population groups (race), 1 to 3.2 for provinces, and 1 to 2.4 for levels of education. Relationship with income and wealth were complex, with highest values for middle income and middle wealth index. The effect of urbanization was small, and reversed in a multivariate analysis. Higher risks in provinces were not necessarily associated with lower income, lower education or higher proportions of home delivery, but correlated primarily with the prevalence of HIV/AIDS.

**Conclusion:**

Demographic census microdata offer the opportunity to conduct an epidemiologic analysis of maternal mortality. In the case of South Africa, the level of MMR increased dramatically over the past 10 years, most likely because of HIV/AIDS. Indirect causes of maternal deaths appear much more important than direct obstetric causes. The MMR appears no longer to be a reliable measure of the quality of obstetric care or a measure of safe motherhood.

## Background

Maternal mortality is an important element of the health transition, and in developed countries the decline in maternal mortality has been dramatic during the 20^th ^century. Beyond the number of maternal deaths, which remains small compared to the number of deaths for all causes combined, maternal mortality bears a strong symbolic value, since these deaths occur "while giving birth". Interest in maternal mortality in developing countries has been gaining momentum since the 1980's with the "Safe Motherhood Initiative", and more recently with the "Millennium Development Goals" (MDG) and the IMMPACT project [1-4]. A recent collection of articles, published in *The Lancet *in 2006, summarizes the demographic and public health debates around maternal mortality [5].

A recurrent issue in assessing maternal mortality levels in developing countries has been the source of data, especially in situations where vital registration is deficient, which is the case for most of Africa [6]. Many attempts were made over the past 50 years to overcome this lack of information. For instance, some of the demographic sample surveys conducted in Africa by the French National Statistical Institute (INSEE) during the 1950s and 1960s included a question on maternal deaths among other questions on deaths in the past 12 months. This was the case with the survey conducted in Burkina Faso in 1960–1961 (Haute Volta at that time), and again later in the same country with the 1991 demographic survey. These surveys were often based on fairly large samples (50,000 to 100,000 households), and produced reliable estimates. With the WFS and DHS programs in vogue since the mid 1970's, the focus became smaller surveys, based on 5,000 to 10,000 households, and a new technique was developed for measuring maternal mortality: the "sisterhood method", with its two variants, the indirect and the direct estimates [7,8]. The sisterhood method measures maternal deaths among the sisters of the respondent, which can therefore increase significantly the number of cases in populations with high fertility. However, if the sample size is small, the number of cases recorded this way is still small, with wide confidence intervals. These are even wider when indirect estimation is used [9]. Furthermore, the sisterhood methods produce estimates for several years before the survey, with imprecise dating in the case of the indirect method. When applied with care, both types of retrospective data provide similar estimates, as shown in a case study in Bangladesh [10].

An alternative is to use a full scale population census, and to investigate maternal deaths in the past 12 months (or 2 to 3 years), to resolve the conundrum of sample size and reference period. Only a few examples are available in Africa: Benin (1992), Madagascar (1993), Lesotho (1986, 1996), Namibia (1991, 2001), Zimbabwe (1992), which have already been reviewed [11], and the South Africa 2001 census, which is investigated in this study. Ours is one of the first studies to use microdata as opposed to published tables. The advantage of microdata is that they may be subjected to a much more detailed analysis, both in terms of data quality and mortality differentials.

The United Nations "Principles and Recommendations for Population and Housing Censuses, Revision 2" recognize the importance of using censuses to measure maternal mortality for countries that lack complete and reliable civil registration and vital statistics systems, even though censuses should not be considered as a substitute for complete and accurate vital registration. The UN principles recommend two follow-up questions in cases where the household being interviewed reports a death during the past 12 months. After ascertaining the name, age and sex of the deceased person and date of death, the interviewer should inquire: 1) Was the death due to an accident, violence, homicide or suicide? 2) If the deceased was a woman aged 15 to 49, did the death occur while she was pregnant, during childbirth, or during the six weeks after the end of pregnancy? [12]. The Africa Addendum to Revision 2 stresses the importance of collecting mortality data, in particular maternal mortality, in the 2010 round of African censuses [13].

In addition to demographic information, many studies have tried to capture maternal mortality from hospital based studies. Of course, these studies have numerous biases, and they may either over-estimate maternal mortality when hospitals function well and attract most of the complicated obstetric cases, or under-estimate maternal mortality when the population has little access to the health infrastructure.

This paper investigates a microdata sample of the 2001 census of South Africa, which included the recommended questions on maternal deaths. Our primary objectives are to estimate maternal mortality levels, and to identify the main sources of differentials through univariate and multivariate analysis. Our secondary objectives are to evaluate the quality of the data and to discuss the relevance of existing definitions of maternal deaths in the context of the HIV/AIDS epidemic.

### Definitions of maternal deaths and pregnancy-related deaths

The World Health Organization (WHO) medical definition of maternal mortality has been stable over the past 30 years: "A maternal death is the death of a woman while pregnant or within 42 days of termination of pregnancy, irrespective of the duration and the site of the pregnancy, from any cause related to- or aggravated by- the pregnancy or its management, but not from accidental or incidental causes" (ICD-10, volume 2, page 134). The WHO definition also distinguishes between the "direct obstetric" causes, resulting from natural obstetric complications or from obstetric interventions, and the "indirect obstetric" causes, resulting from previously existing diseases, or diseases that developed during pregnancy independently from obstetric causes. The ICD-10 makes a special provision for HIV/AIDS and obstetrical tetanus (normally coded among the infectious and parasitic diseases), and recommends to include them among the maternal deaths. In theory, HIV/AIDS should be included in the "indirect cause" category, and the obstetrical tetanus in the "direct cause" category, but this is not explicit in the ICD-10. The ICD-10 manual recommends publishing separately direct causes and indirect causes, however this is rarely done in practice in demographic surveys, or more generally in developing countries, except when the sources of data are medical certificates or special hospital investigations. Note that the strict application of the WHO definition sometimes appears problematic, when highly lethal conditions, such as HIV/AIDS, tuberculosis or acute hepatitis, cause the death of a woman during the maternal period: would the death have occurred at that time if the woman had not been pregnant or delivering?

In demographic censuses and surveys, when causes of death are not available, the practice is to include all deaths during the maternal risk period. The ICD-10 also provides this demographic definition of maternal mortality, labeled "Pregnancy-related deaths", as: "all deaths of a woman while pregnant or within 42 days of termination of pregnancy, irrespective of the cause of death" (ICD-10, volume 2, page 135). In this paper, we considered maternal mortality defined as "pregnancy-related deaths", following the usual practice of demographic surveys and the UN principles and recommendations. Differences from the proper "maternal deaths" are discussed when needed.

Khlat & Ronsmans [14] have shown the difficulty in assessing an attributable risk of maternal mortality from the WHO definition. In fact, during the maternal risk period (40 weeks of pregnancy and 6 weeks post-partum), women appear at the same time at increased risk of death from the direct causes, at lower risk of death from indirect causes for a variety of reasons, and at risk from accident and violence, either related or unrelated with the pregnancy. A simple way of investigating the possible biases is to compare the proportion of deaths due to maternal causes to the proportion of time spent by women in the maternal risk period between age 15 and 49, which depends primarily on the Total Fertility Rate (TFR). If the first proportion is much higher than the second, then the direct causes (or the pregnancy-related violence) are likely to be overwhelming; however, in the case where both are equivalent, positive and negative effects of pregnancy are compensating. We will see such a case when analyzing the 2001 census of South Africa.

## Data and methods

### The IPUMS microdata sample

The 2001 census conducted in South Africa included a question on deaths that occurred in the past 12 months in the household, that is between October 10, 2000 and October 10, 2001. Deaths were recorded by month and year and by age and sex. Furthermore, for each death in the past 12 months, two questions on cause of death were asked: "Did the person die from an accident or through violence?"; "If the deceased was a woman under age 50, did the person die while pregnant, or within six weeks after delivery?". This definition therefore defines maternal mortality as "pregnancy-related death".

The census also included a number of questions which could be used for the analysis of maternal mortality differentials: area of residence (urban/rural), province of residence, level of education, citizenship (nativity), population group (race), ethnicity (defined by the language spoken at home), and religion. In addition, several characteristics of the dwelling unit were also available in the census form (type of living quarters and housing unit, number of rooms, tenure status, source of water, toilet facility, energy or fuel used mainly for cooking, heating, and lighting), as well as a number of household goods (radio, refrigerator, television, telephone, computer, cell-phone). The questionnaire also included an item on annual gross income for each household member, coded in 12 categories, presented as multiples of 4800 ZAR per year, coefficients being 2^n^, with n = 1 to 12.

A 10% sample of the census was entrusted to the IPUMS project by Stats-SA, the Statistical Institute of South Africa, under standard IPUMS access protocols: dissemination to researchers world-wide via the internet without cost. Currently, the IPUMS database  offers samples for 111 censuses encompassing 35 countries and totaling more than 250 million person records. To facilitate comparative research, the IPUMS database offers two types of variables: non-harmonized and integrated. Non-harmonized variables retain the codes and concepts in each set of census microdata. Only for non-response, missing data, blanks and the like, are standard codes applied. Integrated (harmonized) variables are constructed of composite codes, designed to retain all significant detail, yet maintain a common coding scheme for each variable across all censuses and countries. While integration facilitates comparative research, the availability of non-harmonized variables offers a means of checking and correcting errors introduced in the integration process. Some variables, particularly those with subtle differences in definitions, concepts or wording, are available only in non-harmonized form, as is currently the case with mortality questions. For this analysis, survival of mother, household row number of the surviving mother, and delivery in the past 12 months were available only as "non-harmonized" variables. However, with the new release of version 4.0, these and many other variables will become available in integrated form. For details, see the IPUMS web site.

The 2001 census sample includes imputed data, but the original responses are easily derived. Some imputed values were the results of straightforward logical computations, while others were imputed using a standard "hot-deck" procedure. All imputations were performed by Stats-SA, and were identified by means of "flags" in each instance. Of the 59 variables available in the harmonized data set, many had at least some imputed values through hot-decking; among the variables used in this study, maternal deaths, and household income are two key variables with some imputed values [15]. As we shall demonstrate, the imputations were done exceedingly well. In any case, thanks to the use of flags, researchers may check, or re-do, the imputations however they may wish.

### Calculation of maternal mortality levels

Three classic measures of maternal mortality are used in this paper. The maternal mortality ratio (MMR) was calculated as the number of maternal deaths to the number of births in the past 12 months. The number of births in the past 12 months was computed as the number of infants surviving at the time of census backward projected over the past year (further discussed below). The probability of surviving was computed directly from the data (_1_L_0_/S_0_), by calculating the infant death rate (deaths age 0 in past 12 months divided by the infant population at time of census), and applying a separation coefficient (_1_a_0_) of 0.315, derived from model life tables. This calculation was repeated for each group in the differential analysis.

The maternal death rate (MDR) was computed as the number of maternal deaths divided by the female population aged 15–49 at time of census.

The maternal mortality quotient (MMQ), or lifetime risk of dying from maternal causes, was calculated by reconstructing the life table for maternal mortality, by single year of age, from age 12 to age 50 years. Yearly quotients were calculated as the ratio of maternal deaths to the female population at risk, that is the surviving women plus the deaths in the past 12 months.

Statistical testing and confidence intervals were calculated according to formulae provided in standard textbooks [16].

### Socio-economic factors

Most socio-economic variables were used as recoded in the IPUMS integrated sample, which was equivalent to the coding on the census sheet in most cases. Income was given as the mean of the class (for instance 2,400 ZAR for the class ranging from 1 to 4,800 ZAR, etc.), with the exception of the last open class (2,457,601+ ZAR) coded as the bottom value of the class.

The differential analysis presents maternal mortality ratios by socio-economic characteristics taken at the household level, since details at the individual level were not available for maternal deaths. For some factors, the definition of socio-economic characteristics was straightforward, since it applied to all members of the household (place of residence, province). For other characteristics, we took the corresponding value for the head of household (race, ethnicity, nativity). For level of education, we took the highest level in the household. Other types of recoding were tried: level of education of the household head, level of education of women in their reproductive ages, but the highest level in household gave the largest correlations. For income, we cumulated all incomes in the household, and divided by the number of household members, to obtain an income per capita for the household.

We also defined a "wealth index" on the model used by other authors [17]. This index is the sum of 15 dummy variables indicating modern goods in the household or its modern status. The variables selected were: type of dwelling, ownership of dwelling, access to piped water, source of water, toilet facility, fuel for cooking, heating and lighting, refuse disposal, computer, telephone, cellphone, television, refrigerator, and radio.

### Comparing cases and controls

In the multivariate analysis, cases (maternal deaths) were compared with controls, defined as women who were present at the census and who delivered in the past 12 months. This provides all controls in the population, which allows one to compute absolute risks and relative risks, and not only odds ratios as normally done in case-control studies. As for the cases, the characteristics of the household were associated with the controls (and not with the individual characteristics as normally done in case-control studies).

### Analysis of biases

Several biases were investigated. In particular, special attention was paid to the recall period, to age patterns, and to the effect of imputation procedures on the final estimates. The formal analysis of the effect of imputations may be obtained from the authors upon request.

## Results

### Death registration in the census

The IPUMS 10% sample of the 2001 census of South Africa included 36,267 deaths out of a total of 3,725,655 persons enumerated, and 76,292 surviving infants, corresponding to 78,702 births in the past 12 months, with a survival probability of newborns from birth to the census date equal to 0.970. This corresponds to a crude birth rate (CBR) of 21.1 per 1,000 (births/person-years) and a crude death rate (CDR) of 9.7 per 1,000 (deaths/person-years). These values are consistent with other estimates for this period in South Africa.

The distribution of deaths by month and year was quite regular, with an average of 2,790 deaths per month, and a marked seasonality with excess mortality from June to September. However, the month of October was probably over-estimated, since the 21 days of year 2000 (from October 11 to October 31) included 2,641 deaths and the 10 days of year 2001 (from October 1 to October 10) included 2,137 deaths, whereas one would have expected 3,133 deaths in total from the number recorded in the previous and next two months. This suggests that mortality in the previous 12 months was overestimated by some 4.7%, because too many deaths were included in October 2000 (instead of only from October 11 to October 31), and in October 2001 (instead of only from October 1 to October 10).

Of the 8,236 deaths of females aged 12–50 in the sample, 18.2% had no information on whether the death was maternal or not. Using the hot-deck method, Stats-SA imputed 116 blank records as pregnancy-related (out of a total of 508) and 1,383 as not pregnancy-related (out of a total of 7,728). All imputations are flagged. We exhaustively analyzed these imputations and concluded that the hot-decking showed no sign of bias by household characteristics. However, because the age pattern of maternal mortality was not perfectly matched, too few maternal deaths were imputed below age 20, and too many above age 38. As a result, the number of maternal deaths appeared inflated by 5.9%. The effect is small, and strengthens our confidence in the analysis which follows.

### Maternal mortality level

The IPUMS sample of the 2001 census of South Africa included 508 maternal deaths, out of 1,048,824 women aged 15–49 years. Straightforward calculations give a maternal mortality ratio (MMR) of 646 per 100,000 live births, a maternal death rate (MDR) of 48.4 per 100,000 women, and a lifetime risk (MMQ) of 1,681 per 100,000 (Table 1). Matching the MMR and MMQ corresponds to a total fertility rate (TFR) of 2.62, which is basically equal to the TFR expected from the two previous DHS surveys (TFR = 4.58 at the 1988 DHS for the 1985–1988 period, and TFR = 3.10 at the 1998 DHS for the 1995–1998 period predict a TFR of 2.60 in 2001). These estimates are therefore internally consistent.

**Table 1:** Maternal mortality indicators estimated from the 2001 census (10% sample), South Africa

		95% confidence interval	
Maternal mortality indicators	Value	Min	Max
*Raw estimates*			
Maternal mortality ratio (MMR), per 100,000 live births	646	592	705
Maternal death rate (MDR), per 100,000 person-years lived	48.4	44.4	52.8
Life time risk of maternal mortality (MMQ) per 100,000 women at age 12	1681	1541	1833
			
*After correcting for biases*			
Maternal mortality ratio (MMR), per 100,000 live births	542	497	591

Sources: Statistics South Africa. *Ten Percent Sample of the 2001 Census*. Pretoria, 2006. Minnesota Population Center. *Integrated Public Use Microdata Series *– International: Version 3.0. Minneapolis, University of Minnesota, 2007.

Note: Correction for biases: see text for details

Of these 508 pregnancy-related deaths, 78 were also due to accident and violence (15%), a proportion that is high by African standards, and higher than for non-maternal deaths (10%), most likely because of the age structure of maternal mortality, and possibly because of an excess of these deaths associated with pregnancy. This high proportion did not seem to be due to imputations, since it was similar for non-imputed deaths (14.3%). The proportion of direct causes, indirect causes, and of causes unrelated with the pregnancy remains unknown in the census.

The value of the MMR may appear high for a country such as South Africa, even considering the demographic definition of maternal mortality. However, the total number of deaths of females age 15–49 in the 12 months preceding the survey was 7,934, which reduces maternal deaths to only 6.4% of the total. With a TFR of 2.62 children per woman, some 6.6% of the time of women aged 15–49 is spent in the maternal risk period (2.62 times 46 weeks in the maternal risk period, out of 35 years spent between age 15 and 50), which means that the observed proportion of maternal deaths is equivalent to that expected from the level of mortality in the population. As a consequence, the deaths attributable to obstetric causes must be compensated by the lower risk of pregnant women from indirect causes, for a variety of reasons that remain to be explored. In conclusion, it is because female adult mortality is extremely high in South Africa that the MMR also appears very high.

The female mortality quotient between ages 15 and 50 years (_35_q_15_) calculated from the 2001 census data was 0.2471, which corresponds to a life expectancy of 49.7 years in the UN model life table system for developing countries (general pattern). This is obviously much lower than the observed female life expectancy in the census data (e° (0) = 64.4 years). This discrepancy is due to the fact that the mortality level at young adult ages is much higher than that at other ages, in particular because of the high burden of HIV/AIDS and of external causes. Mortality below age 15 (_15_q_0_) was 0.072, which corresponds to a life expectancy of 68.1 years in model life tables. Life expectancy above age 50 was 30.7 years, which corresponds to a life expectancy of 77.1 years in model life tables. Taking an average of 72.6 years as a reference value for life expectancy, adult female mortality in the census appeared as 4.7 times higher than expected from model life tables based on other age groups (expected _35_q_15 _= 0.05237). If this coefficient is applied, the MMR would be only 137 per 100,000.

The 2001 census estimates for the Shangaan living in rural areas of the Limpopo province could be compared with the Agincourt DSS, a population from the same ethnic group living in the same province, and which accounts for about 7% of the total Shangaan living in Limpopo [18,19]. Maternal mortality in Agincourt is also primarily defined as "pregnancy-related deaths", although the Agincourt DSS includes causes of deaths assessed by verbal autopsies which allow for further analysis. The MMR for the 2000–2002 period in Agincourt was 305 per 100,000 live births (15/4912), not significantly different from the MMR among the Shangaan from rural Limpopo at the 2001 census (MMR = 382 per 100,000; P = 0.593). Note that the two populations were also comparable in birth rates (24 per 1,000 in both cases, P = 0.970), and in life expectancy at birth for men (e° = 58.7 and 57.1 respectively, P = 0.251). However, female mortality in 2000–2002 was higher in Agincourt than at the census (e° = 64.3 and 72.9 respectively, P < 0.001), and this was true for all adult age groups. This is probably due to a higher level of HIV/AIDS infections among women in Agincourt than among the other Shangaan people of Limpopo, since female life expectancy averaged also 72 years in 1992–1994 in Agincourt, before HIV/AIDS became an important cause of death.

In-depth studies on long-term trends of maternal mortality were conducted in the Cape Town Peninsula, from births and deaths data recorded in hospitals [20-22]. Results show first a marked decline in MMR from 301 per 100,000 in 1953 to 31.2 per 100,000 in 1987–1989, followed by a marked increase reaching 112 per 100,000 in 2002. These data are not strictly comparable to the census data, since they apply to the most developed part of the province, and are based on hospital data and on the medical definition. However, they indicate firstly that even in the most advanced part of the country, MMR was already high (112 versus 306 for the whole province at the 2001 Census), and secondly, that the MMR has soared by a factor of 3.6 in the recent years, mostly because of indirect causes, in particular HIV/AIDS, and to a lesser extent to hypertension and pregnancy-related sepsis.

Another element corroborating the MMR estimates is given by hospital statistics on induced abortion. Dickson and Rees [23] report that "425 women died in hospitals each year from complications of unsafe, clandestine abortions". The confidential enquiry on maternal deaths [24] estimated that out of 133 cases of maternal deaths investigated, 12 were due to septic abortion, out of which 7 were due to induced abortion outside of health services. Even if these data apply to a time when the "Termination of Pregnancy Act" was just passed, it gives an order of magnitude of the number of maternal deaths from other causes. Applying the ratio of 133/12 to the 425 abortion deaths gives 4,710 maternal deaths, a figure consistent with the 5,080 deaths extrapolated to the whole population from the 10% sample.

### Fine-tuning the maternal mortality level

The raw estimates of maternal mortality presented above included several biases. Two were already mentioned for the numerator: inclusion of too many deaths during the 12 months period, and too many maternal deaths that were imputed. The denominator used could also be questioned. The number of births backward projected appears somewhat low. The number of women who delivered in the past 12 months (92,238) appeared too high, much higher than in the previous two years, and could not be readily used. From women who last delivered in the past three years (238,596), we estimated the mean annual number of births to be 84,520, after correcting for multiple births, and for women who had more than one delivery over the three-year period. This number is consistent with the number of registered births in the whole population (928,670 after inclusion of late registration), assuming a 10% census undercount compared with vital registration. Taking into account all these minor biases, the final estimate for MMR becomes 542 per 100,000 live births. According to this figure, there were some 5,000 "pregnancy-related deaths" in South Africa in 2001 (which is basically 10 times the raw number found in the 10% sample of the census before corrections...). This final estimate is also consistent with the 575 per 100,000 figure estimated by Dorrington and colleagues from the vital registration. [personal communication of Dr. R. Dorrington]. Note that only a fraction of those qualify as "maternal deaths" according to the medical definition, and even a smaller fraction as "direct obstetric causes".

### Maternal mortality differentials

South Africa is a complex society, with sharp gradients by race and level of economic development. Some groups approach European living standards, whereas others are closer to those of remote areas of sub-Saharan Africa. As a consequence, mortality differentials are usually very large, and this applies to maternal mortality as well. In addition to living standards, mortality levels and trends are also compounded by the raging HIV/AIDS epidemic and by the high mortality from accidents and violence. Both groups of causes of death maintain a complex relationship with socio-economic status, often different from that of other causes of death, such as other infectious and parasitic diseases or non-communicable diseases. We present here only differentials in the MMR, since the relationships with the other maternal mortality indicators are similar (Table 2).

**Table 2:** Differentials in maternal mortality ratio, South Africa 2001 census.

				95% Confidence interval		
					
Variable	Category	Maternal deaths (N)	MMR, per 100,000 live births	Min	Max	Relative risk
Total	Total	508	551	505	601	
Residence	Urban	249	505	446	572	1.00
	Rural	259	604	535	682	1.20
Province	Kwazulu Natal	152	772	659	905	3.15
	North-West	57	751	579	974	3.07
	Eastern Cape	88	712	578	877	2.91
	Free State	34	654	467	915	2.67
	Mpumalanga	37	519	376	716	2.12
	Gauteng	7	415	198	871	1.69
	Northern Cape	70	395	313	499	1.61
	Limpopo	41	347	256	471	1.42
	Western Cape	22	245	161	372	1.00
Race	Black/African	478	614	561	672	9.16
	Coloured	24	284	190	424	4.24
	Indian/Asian	3	200	65	620	2.99
	White/European	3	67	22	208	1.00
Language	IsiZulu	183	759	657	877	7.23
	IsiXhosa	114	683	568	821	6.50
	SeSotho	47	642	482	854	6.11
	SeTswana	50	634	481	837	6.04
	IsiNdebele	8	541	271	1082	5.15
	XiTsonga	22	449	296	682	4.28
	SePedi	39	426	311	583	4.06
	SiSwati	12	416	236	733	3.96
	TshiVenda	2	81	20	324	0.77
	Afrikaans	24	236	158	352	2.25
	English	5	105	44	252	1.00
Education	0–4 years	52	899	685	1180	2.35
	5–8 years	121	753	630	900	1.97
	9–11 years	183	598	517	691	1.57
	12+ years	152	382	326	448	1.00
Income	None declared	142	650	551	766	3.13
	< 2400 Rd	181	587	507	679	2.82
	2400–4800 Rd	96	674	552	823	3.24
	4800–9600 Rd	52	482	367	633	2.32
	9600–28800 Rd	25	286	193	423	1.38
	> 28800 Rd	12	208	118	366	1.00
Wealth index (nb items)						
	0	12	899	511	1583	1.20
	1	23	524	348	789	0.70
	2	37	536	388	740	0.71
	3	38	497	362	683	0.66
	4	51	672	511	884	0.90
	5	46	651	488	869	0.87
	6 (ref.)	49	750	567	992	1.00
	7	43	695	515	937	0.93
	8	40	657	482	896	0.88
	9	41	661	487	898	0.88
	10	29	477	331	686	0.64
	11	29	452	314	650	0.60
	12	36	505	364	700	0.67
	13	23	353	235	531	0.47
	14	10	249	134	463	0.33
	15	1	47	7	334	0.06

The MMR was only marginally higher in rural areas (604 per 100,000) than in urban areas (505 per 100,000). This is probably due to the balance between diverging forces: more HIV/AIDS and more external causes in urban areas, and less access to medical services in rural areas (Table 2).

**Figure 1:**
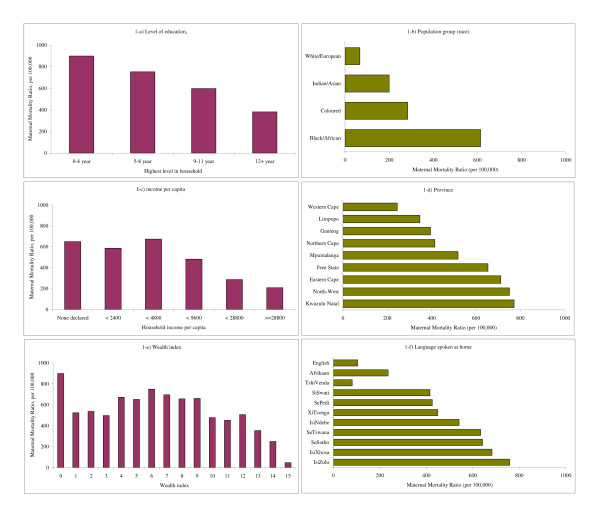
**Maternal mortality differentials, South Africa, 2001 census**. Note: Vertical bars are used for quantitative variables. Horizontal bars are used for qualitative variables, after ordering from low to high.

The gradient by province was very marked, from 3.2 to 1 between the province with highest maternal mortality (Kwazulu-Natal, 772 per 100,000) and the province with the lowest MMR (Western-Cape, 245 per 100,000) (Figure 1-d). South African provinces vary very much in their ethnic composition, as well as in their level of development. Kwazulu-Natal is the most populated, has the highest HIV seroprevalence rates and the lowest life expectancy, but fares better in terms of education, income and wealth. On the other side of the spectrum, Western Cape has the lowest HIV seroprevalence rates, the highest life expectancy, the highest level of education, the highest wealth index, and the next-highest income. The wealthiest province (Gauteng) has a MMR below average, but still somewhat higher than the MMR in the poorest province (Limpopo). These two contrasting provinces have similar life expectancy (67.6 and 66.4 respectively), similar levels of female adult mortality at age 15–49 (0.155 and 0.172 per 1,000), but Gauteng has higher HIV infection rates (29.8%) than Limpopo (14.5%). These probably explain the differences in maternal mortality, since the death rates from external causes were about the same.

The gradient by population group (race) was even more marked, with a range from 9.2 to 1 from Black/African (614 per 100,000) to White/European (67 per 100,000), the two other groups being in an intermediate situation: Coloured: 284 per 100,000, and Indian/Asian: 200 per 100,000 (Figure 1-b).

The gradient by ethnicity, measured by the language spoken at home, reflects at the same time race, urbanization, and location. As could be anticipated, the range of variation was also considerable, from 1 to 7.2 (Figure 1-f). The two groups speaking European languages appeared in a more favorable situation: speakers of English (105 per 100,000) and Afrikaans (236 per 100,000). Among the speakers of African languages, the levels of MMR were much higher, with a range from 416 per 100,000 (SiSwati) to 759 per 100,000 (IsiZulu), with the exception of the TshiVenda (81 per 100,000). This group accounted for only two maternal deaths, therefore the confidence interval is huge (20 to 324 per 100,000), although still significantly lower than the next lowest African group, the SiSwati (P = 0.032).

The gradient by level of education was small, compared with gradients by province, primarily because of the high level of education in the country, even in remote rural areas (Figure 1-a). The range was from 1 to 2.4 between 12+ years of education (MMR = 382 per 100,000) and less than 5 years of education (MMR = 899 per 100,000).

The gradient by level of income was complex, with two peaks, one for the very poor, who declared no monetary income (MMR = 650 per 100,000), and the other for the intermediate category (2400–4800 ZAR per capita), with basically the same MMR (674 per 100,000) (Figure 1-c). The differences between contiguous categories for groups above the intermediate category were statistically significant (P = 0.052 between III and IV, and P = 0.032 between IV and V), whereas differences between contiguous categories for groups below the intermediate were not (P = 0.363 between I and II, P = 0.274 between II and III). Thus, maternal mortality was not uniform for lower income groups below the intermediate category. This seems to be due to the complex relationships between area of residence, income, HIV/AIDS and level of mortality from all causes.

The gradient by level of wealth, measured by the wealth index, was even more striking than the income gradient (Figure 1-d). The relationship had a complex shape, with ups and downs, with lower levels of MMR for the fourth category (3 items: MMR = 497 per 100,000) and the wealthiest (15 items: MMR = 47 per 100,000), and the maximum for the very poor (no item: 899 per 100,000) and the intermediate category (6 items: 750 per 100,000).

The correlations between the MMR and the various variables at the provincial level revealed the key factors. Correlation between the MMR and any indicator of mortality was very high, and reached -0.91 with life expectancy, and was also high (+0.73) with the prevalence of HIV in the province. Correlations with indicators of socio-economic status were average: -0.42 with education; -0.49 with income, -0.49 with wealth, -0.32 with urbanization. Relationships with racial composition were complex: negatively related with the proportion of White/European (-0.53) and Coloured (-0.58), but positively related with the proportion of Black/African (+0.58) and Indian/Asian (+0.45), the last being explained by the concentration of Indian/Asian in Kwazulu-Natal. Correlations with fertility (+0.14) and with population density (-0.15) were very small. Correlation with the proportion of home deliveries was positive, as expected, but of small magnitude (+0.29), underlying again the importance of indirect causes in maternal mortality levels in South Africa.

### Multivariate analysis

Because of the complex social fabric of South Africa and the heavy presence of HIV/AIDS, the multivariate analysis is particularly difficult to interpret. A series of linear-logistic regression models were run on cases (maternal deaths) versus controls (surviving women), which provide not only odds ratios, but also absolute risks since controls cover the whole population. However, it should be noted that the analysis is not strictly equivalent to a formal case-control study, since individual characteristics are not known for women who died, but only the characteristics of their households. In addition to household characteristics, we have added, in a second model, some of the characteristics of the province (community variables) likely to have an effect on maternal mortality ratios, namely the HIV seroprevalence rate in 2001, the proportion of home delivery births, and the female death rate from external causes at age 15–49 years.

Examining household characteristics revealed some interesting features, when controlling for urban residence, education, race, wealth, and province (Table 3). Firstly, the effect of urban residence changed, with higher levels of MMR in urban areas (+28% instead of -16% in the univariate analysis). Secondly, the effect of the level of education became even smaller, with a net effect of -17% for one standard deviation (3.2 years of schooling). Relationship with income or wealth was not linear, and their net effect in the logit-linear regression were not significant. As seen above in the univariate analysis, the relationship with wealth was quadratic, and better seen as a peak for medium values of wealth (6-items). We therefore coded a "wealth distance" as the absolute difference between the number of items in the household and the medium value. Taken this way, the effect of wealth was significant, but remained relatively small, and of similar magnitude as that of education. The ranking of the four racial groups was confirmed by the multivariate analysis. Large differences were also seen within provinces, keeping basically the same relationships as in the univariate analysis. The difference between Limpopo and Western Cape even disappeared in the multivariate analysis, because the small difference noted in the univariate analysis was compensated by the effect of large differences in income and education. The case of Limpopo deserves further investigation, not only for maternal mortality, but also for overall mortality, which appears lower than expected from its level of socio-economic development. Note that the Agincourt DSS, located in Limpopo, confirms these findings.

**Table 3:** Risk factors of maternal mortality at the household level, South Africa, 2001 (from linear logistic regression)

Household characteristics	Beta	Standard error	Net effect	Relative risk	P-value	Signif.
Level of education	-0.0592	0.0131	287	0.83	0.0000	*
Wealth distance	-0.0651	0.0231	298	0.86	0.0048	*
Urban areas	0.2477	0.1076	444	1.28	0.0213	*
Black/African (ref)			347	1.00		
Coloured	-0.3624	0.2520	242	0.70	0.1504	
Indian	-0.9890	0.5889	129	0.37	0.0931	
White	-1.6661	0.5859	66	0.19	0.0045	*
Western Cape (Ref)			347	1.00		
Eastern Cape	0.8304	0.2709	792	2.28	0.0022	*
Northern Cape	0.3743	0.4357	504	1.45	0.3902	
Free State	0.6608	0.3017	670	1.93	0.0285	*
Kwazulu Natal	0.9320	0.2664	876	2.53	0.0005	*
North-West	0.8292	0.2857	791	2.28	0.0037	*
Gauteng	0.2955	0.2745	466	1.34	0.2818	
Mpumalanga	0.4790	0.3023	559	1.61	0.1131	
Limpopo	0.0882	0.3050	379	1.09	0.7724	
Constant	-5.0586	0.2911	347			

N.B. Reference categories: Race = Black/African, Province = Western Cape. Net effects are calculated for dummy variables, and for one standard deviation for quantitative variables (education and wealth). Education is measured in years of schooling. Wealth distance is counted as the distance in the wealth index from the average (6 items).

In order to better approach the large differences between the provinces, further controls were added: HIV prevalence in the province, published by the Ministry of Health for 2001 (sentinel sites of pregnant women); death rate at 15–49 from external causes, calculated from the 2001 census data; and proportion of home deliveries, taken from the 1998 DHS survey. Results confirmed the probable role of HIV/AIDS and external deaths in the overall level of maternal mortality: both effects were positive and significant (Table 4). The effect of the home delivery variable, however, was not significant, and even somewhat negative, contrary to what was expected. This indicates that indirect causes, and in particular HIV/AIDS and external causes, and possibly other non-maternal causes, are the most important factors for determining MMR levels, and that conversely direct causes play a much smaller role in the differentials.

**Table 4:** Risk factors of maternal mortality at the household and provincial level, South Africa, 2001 (from linear logistic regression)

Household characteristics	Beta	Standard error	Net effect	Relative risk	P-value	Signif.
*Provincial level*						
HIV prevalence	0.0173	0.0082	689	1.15	0.036	*
Violence death rate	0.0393	0.0088	795	1.33	0.000	*
Home delivery	-0.0072	0.0085	566	0.95	0.399	
*Household level*						
Level of education	-0.0588	0.0131	497	0.83	0.000	*
Wealth distance	-0.0638	0.0228	517	0.86	0.005	*
Urban	0.2103	0.0993	738	1.23	0.034	*
Black/African (ref)			599	1.00	0.000	*
Coloured	-0.3925	0.2379	405	0.68	0.099	
Indian	-0.9613	0.5872	230	0.38	0.102	
White	-1.6767	0.5858	113	0.19	0.004	*
Constant	-5.9427	0.3092	599			

## Discussion

The census has a great potential for monitoring levels, trends and differentials in maternal mortality. By definition, the census provides a complete picture of the whole population, and therefore avoids issues of representativeness which often hamper estimates based on medical statistics. The census also offers large numbers and small confidence intervals, in contrary to demographic surveys with small sample size. It also permits precise point estimates, which is not the case for estimates derived from the survival of sisters, an important feature in situations where changes are rapid. Census microdata facilitate a variety of univariate and multivariate analyses at the household level, which can reveal the source of major differentials in a country. Even if less precise than formal case-control studies investigating the effect of individual characteristics on maternal mortality, and if the list of variables is small and imposed by the census, the lessons learned still appear important for public health professionals.

The quality of the estimates of mortality and fertility derived from the 2001 census have been discussed by other authors [25]. It is beyond the scope of this paper to enter into a full discussion of the biases involved, or to attempt at correcting the estimates. Our approach has been to stick to the internal consistency of the data, and to the external consistency with other independent sources. However, it should be remembered that some of the estimates may be off by 10% or more, and should therefore be considered with caution.

Levels for MMR in 2001 South Africa appear much higher than previous estimates [26,27]. The 1998 DHS estimate (150 per 100,000 live births) covered a different period (1992–1997), when HIV/AIDS was not yet a major cause of death, and was based on a tiny sample (19.2 deaths after sampling weights were applied) that produce a large confidence interval, to which should be added the variance due to the sampling technique, and another variance due to the sisterhood method. According to the 2001 census, the MMR was 4.3 times higher, the MDR was 3.3 higher, and the GFR was 0.77 times lower. Even taking into account the large confidence intervals of the 1998 DHS, there is no doubt that maternal mortality has been increasing dramatically in South Africa over the past 10 years, which is confirmed by local studies in Cape Town and in Agincourt.

The proportion of maternal deaths among deaths of women in their reproductive ages appears much lower than that observed in other countries with similarly high levels of MMR. For instance, in Niakhar, Senegal, the MMR was 519 per 100,000 in 1983–1989, with a proportion of maternal deaths equal to 26.3% [28]. In Nouna, Burkina Faso, the MMR was 389 per 100,000, and the proportion of maternal deaths was 24.1% [29]. In Matlab, Bangladesh over the 1976–1985 period, the MMR was 551 per 100,000, accounting for 37.3% of all deaths of women aged 15–44 years [30]. In the case of South Africa, the proportion of maternal deaths was only 6.6%, closely approximating the proportion of time spent in the maternal risk period. Assuming that half of the maternal deaths were due to direct causes implies that women are at a much lower risk from indirect causes during the maternal risk period. Of course, a proper investigation of direct and indirect causes should be done to better understand this phenomenon, and in particular by considering separately cases of premarital fertility (very young women), middle-aged women, and older women, who have very different obstetric risks, and are differentially affected by HIV/AIDS and by pulmonary tuberculosis [31].

Maternal mortality differentials appeared quite distinct from classic differentials observed elsewhere in Africa or in Asia [32]. Above all, the ethnic/racial and provincial differences were overwhelming in South Africa, even after controlling for socio-economic status. Furthermore, relationships with income and wealth were more complex than elsewhere. This has also been observed in Agincourt, where mortality from infectious diseases other than HIV/AIDS were negatively related to socio-economic status, but where mortality from chronic diseases, from accident and violence, and from HIV/AIDS and PTB were either inversely related, or had a U-shape relationship. Lastly, the association with urbanization was also different from that expected, with a small gradient, that was even reversed in the multivariate analysis. A similar observation was made in the hospitals of Kwazulu-Natal [26]. These patterns could be understood within the framework of the complex social fabric of South Africa, and its recent history.

The magnitude of the changes noted in maternal mortality in South Africa is simply astonishing, and calls for a careful monitoring of trends and patterns. Vital registration has improved dramatically in South Africa from 1991 to 2001, complicating trend analysis. However, its coverage is becoming high and stable for adults, and it can now be used for monitoring trends in maternal mortality in the coming years. This will provide at least trends in direct causes, the most likely to be picked up in the medical certificates.

Another important finding of this study is the major role played by indirect causes. This makes the analysis of MMR using the demographic definition of maternal mortality more difficult than before. The main aim of the MMR was to target the obstetric causes, with the idea of improving the safety of pregnancies and deliveries. We may now be in a situation where direct causes continue to decrease while indirect causes increase, so that trends in MMR reflect primarily trends in indirect causes. This changes totally the interpretation of the MMR, and its implication for monitoring progresses in safe motherhood.

One of the Millennium Development Goals (MDG) is to reduce maternal mortality by three fourths by 2015. In order to monitor progresses made in obstetric care, new analytical tools are needed, since the MMR obtained when using the demographic definition (pregnancy-related deaths) is unlikely to be appropriate in cases where emerging diseases and external causes play an increasing role. There is a need to go beyond the demographic numbers, and to look more carefully at trends in direct obstetric causes of death in developing countries. If the census provides the numbers, it should be complemented with cause of death information. This can be done by full scale verbal autopsies, detailing the timing of the death (early pregnancy, late pregnancy, delivery, post-partum period, induced abortion), the leading obstetric causes (hemorrhage, eclampsia, obstructed labor, pulmonary embolism, post-partum infections, etc), the leading indirect causes (HIV/AIDS, PTB, hepatitis, etc.), and the leading external causes (road traffic accidents, household accidents, homicide, suicide, etc.). Verbal autopsies have been used with success for a long time for investigating maternal deaths, and are likely to add immensely to the whole picture [28,30-35].

## Conclusion

Our study leads to the recommendation of applying fully the WHO guidelines for maternal mortality, in particular to separate obstetric causes from indirect causes. This is the only way to measure simultaneously progress made with the Safe Motherhood Initiative, and to evaluate rising costs due to HIV/AIDS and other emerging or resurgent diseases.

## Conflict of interests

The authors declare that they have no competing interests.

## Authors' contributions

MG conducted the data analysis and wrote the first draft of the paper. RM initiated the study, and contributed to the analysis and to the writing of the final draft. KM contributed to the writing and provided the key references from the United Nations. All authors have read and approved the final manuscript.
